# NDRG2 regulates the formation of reactive astrocyte-derived progenitor cells *via* Notch signaling pathway after brain traumatic injury in rats

**DOI:** 10.3389/fnmol.2023.1149683

**Published:** 2023-04-04

**Authors:** Qinjun Zhang, Rui Shi, Minghua Hao, Dongyun Feng, Rui Wu, Ming Shi

**Affiliations:** ^1^Department of Neurology, Xijing Hospital, Fourth Military Medical University, Xi’an, Shaanxi, China; ^2^Department of Neurology, Meishan Cardio-Cerebrovascular Disease Hospital, Meishan, Sichuan, China; ^3^Department of Neurology, Shandong Armed Police General Hospital, Jinan, Shandong, China

**Keywords:** NDRG2, Notch signaling, astrocyte, Rad-PCs, traumatic brain injury, rat

## Abstract

In response to traumatic brain injury, a subpopulation of cortical astrocytes is activated, resulting in acquisition of stem cell properties, known as reactive astrocytes-derived progenitor cells (Rad-PCs). However, the underlying mechanisms remain largely unknown during this process. In this study, we examined the role of N-myc downstream-regulated gene 2 (NDRG2), a differentiation- and stress-associated molecule, in Rad-PCs after cortical stab injury in adult rats. Immunohistochemical analysis showed that in the cerebral cortex of normal adult rats, NDRG2 was exclusively expressed in astrocytes. After liu cortical injury, the expression of NDRG2 was significantly elevated around the wound and most cells expressing NDRG2 also expressed GFAP, a reactive astrocyte marker. Importantly, NDRG2-expressing cells were co-labeled with Nestin, a marker for neural stem cells, some of which also expressed cell proliferation marker Ki67. Overexpression of NDRG2 further increased the number of NDRG2/Nestin double-labeling cells around the lesion. In contrast, shRNA knockdown of NDRG2 decreased the number of NDRG2^+^/Nestin^+^ cells. Intracerebroventricular administration of stab-injured rats with a Notch antagonist, DAPT, led to a significant decrease in Nestin^+^/NDRG2^+^ cells around the injured boundary, but did not affect NDRG2^+^ cells. Moreover, overexpression or knockdown of NDRG2 led to up- and down-regulation of the expression of Notch intracellular domain NICD and Notch target gene Hes1, respectively. Taken together, these results suggest that NDRG2 may play a role in controlling the formation of Rad-PCs in the cerebral cortex of adult rats following traumatic injury, and that Notch signaling pathway plays a key role in this process.

## Introduction

Traumatic brain injury (TBI) is a major global public health concern, which affects millions of people worldwide (Sande and West, 2010). With advances in treatment options, the mortality rate of TBI is greatly decreased, but the high incidence of severe disabilities and cognitive impairment remains a huge public health concern. Furthermore, clinical strategies focused on improving brain functional recovery are still lacking. The TBI-induced functional deficits usually result from neuronal, axonal and myelin loss, reactive gliosis and neuroinflammation (Raghupathi, 2004; Iverson, 2005). Although various strategies of pharmacotherapy, rehabilitation, or neuropsychology have been used to ameliorate a range of behavioral and cognitive impairment (Liepert, 2016; Azouvi et al., 2017; Dang et al., 2017), these therapeutic methods failed to demonstrate meaningful efficacy. This has prompted researchers to seek alternative strategies for treating TBI.

Several lines of evidence suggest that mature mammalian brain harbors neural stem cells (NSCs) with regenerative potency following brain insults (Dayer et al., 2005; Thored et al., 2006; Yamashita et al., 2006; Hou et al., 2008; Blaiss et al., 2011; Sun et al., 2015). Two types of NSCs have been identified in the adult brain: endogenous NSCs from neurogenic niches and injury-induced NSCs from brain parenchyma. The endogenous NSCs are primarily located in the subventricular zone (SVZ) of the lateral ventricle and the subgranular zone (SGZ) in the dentate gyrus (DG) of the hippocampus (Altman and Das, 1965; Lois and Alvarez-Buylla, 1993). NSCs from the SVZ can generate new neurons, which migrate along with rostral migratory stream to olfactory bulb and become olfactory granule or periglomerular neurons while those from the DG can migrate from the SGZ to the granular cell layer and differentiate into DG granule cells (Gritti et al., 2002; van Praag et al., 2002; Sun, 2016; Sailor et al., 2017). When various insults occur in the brain, endogenous NSCs, e.g., those from SVZ, shift from their routine migratory path into the injured regions, such as striatum and cerebral cortex, where only a small number of NSCs can survive and differentiate into functional neurons (Arvidsson et al., 2002). Apart from endogenous NSCs, recent studies have showed that in response to brain injuries such as stroke or TBI, subpopulations of local astrocytes in the brain parenchyma can be activated and then acquire properties of neural stem cells, known as reactive astrocytes-derived neural stem/progenitor cells (Rad-PCs) (Buffo et al., 2008; Shimada et al., 2012; Gotz et al., 2015). These injury-induced progenitor cells could form multipotent spheres in culture (Buffo et al., 2008) and differentiate into astrocytes and oligodendrocytes (Shimada et al., 2012), or into functional neurons when forced expression of transcription factor NeuroD1 (Guo et al., 2014). Therefore, these *in situ* Rad-PCs appear to have a significant latent capacity, which is gaining increasing attention, and may provide a source of multipotent cells for cell transplantation strategy, probably benefiting brain functional recovery after TBI. However, the molecules that govern the formation of Rad-PCs in cortical parenchyma remain largely unknown.

N-myc downstream-regulated gene 2 (NDRG2), previously known as a tumor suppressor molecule (Deng et al., 2003), is involved in cell differentiation and stress-associated events (Ma et al., 2017). In the central nervous system, NDRG2 is primarily expressed in astrocytes in brain parenchyma under the normal condition (Okuda et al., 2008; Takeichi et al., 2011; Flugge et al., 2014). Its expression is upregulated when brain is subjected to various insults such as ischemia, hemorrhage, or trauma (Li et al., 2011; Takarada-Iemata et al., 2014; Gao et al., 2018), suggesting that NDRG2 may exert neuroprotective effects, e.g., maintaining the integrity of blood brain barrier (Takarada-Iemata et al., 2018) and enhancing the clearance of glutamate in synaptic cleft (Yin et al., 2020). Apart from its expression in parenchymal astrocytes, we have shown that NDRG2 is abundantly expressed in proliferating cells in the neurogenic germinal regions of both embryonic and post-natal mouse brains (Liu et al., 2012), indicating that NDRG2 may also play a crucial role in neurogenesis of endogenous NSCs. However, whether NDRG2 is involved in injury-induced progenitor cells after TBI was still unknown. To this end, we investigated potential regulatory effects of NDRG2 on Rad-PCs in adult rats after cortical stab injury in this study, and showed that NDRG2 may play a role in controlling the formation of injury-induced progenitor cells by regulating Notch signaling pathway.

## Materials and methods

### Animals

Adult male Sprague-Dawley (SD) rats, aged 3−4 months and weighed 250−300 g, were obtained from the Laboratory Animal Centre of the Fourth Military Medical University (Xi’an, China). Before the experiments, SD rats were housed with food and water available *ad libitum* in an air-conditioned room. Animal experiments were approved by the Fourth Military Medical University Animal Experimentation Committee, and conducted under the ethical guidelines in compliance with the National Institutes of Health Guidelines for the Care and Use of Laboratory Animals.

### Adenovirus production

For overexpression of NDRG2, a full-length coding sequence of rat *ndrg2* gene (GenBank No.: NM_001270862) was subcloned and inserted into an adenoviral vector to generate pAd-NDRG2. Using an adenovirus expression kit (Genechem, Shanghai, China), viral particles were packaged in HEK 293 cells to generate recombinant adenoviruses encoding *ndrg2* (Ad-NDRG2). For knockdown of NDRG2 expression, the sequence containing *ndrg2* short hairpin RNA (shRNA) was inserted into a recombinant adenoviral vector (Genechem, Shanghai, China) to generate pAd-NDRG2-shRNA. The sequence of the shRNA for *ndrg2* was designed based on its coding sequence, and the optimal shRNA sequence for knockdown (5′-GGGGATATGCAAGAGATCATA-3′) was selected. The scrambled sequence was used as the negative control (5′-GTTCTCCGAACGTGTCACGTA-3′). Adenoviruses encoding *ndrg2*, *ndrg2*-shRNA, and *ndrg2*-scramble were amplified in HEK293 cells. Viral titers were determined by a plaque-forming assay in HEK 293 cells. Viral stocks had titers of more than 1 × 10^9^ PFU/ml. The efficiency of adenoviral-mediated upregulation or knockdown of NDRG2 expression was tested in HEK293 cells and semi-quantified by immunoblot analysis (Supplementary Figure 1).

### Cortical stab injury

Cortical stab injury was performed as previously described (Auguste et al., 2007; Buffo et al., 2008; Robel et al., 2011), with slight modifications. Briefly, adult male SD rats were anesthetized with chloral hydrate (400 mg/kg, i.p.) and then placed in a stereotaxic device. After the head skin was incised using a scalpel blade, a hole was drilled on the right hemisphere at approximately 2.2 mm right of the midline and 0.4 mm posterior to the Bregma using a dental drill. A stab wound lesion was created by inserting a 26-gauge needle to the right cerebral cortex at a depth of 1.8 mm. The sham control rats were only drilled on the skull but not subjected to stab injury. At 1 d, 3 d, 5 d, and 7 d after the stab injury, the rats were sacrificed and subjected to the morphological and immunoblotting analyses described below. For each set of comparison, at least three experimental and control rats were included.

### Adenovirus injection

Immediately after stab injury, adenoviruses containing NDRG2 overexpressing (Ad-NDRG2) or knockdown (Ad-NDRG2-shRNA) constructs were injected with a 26-gauge needle. For the control, the adenoviral blank (Ad-vector) or scramble (Ad-SC-shRNA) vector was also injected. In addition, in order to exclude the possible effects of adenoviruses themselves on experiments, same volume of saline was also injected. The injection volume and rate were at 8 μl at 0.5 μl/min, and the needle was gradually moved up during injecting at a speed of 0.1 mm/min. After injection, the needle was maintained for at least 5 additional minutes and then slowly withdrawn. After the surgery, the animals were kept warm in isolation for 3 h until complete recovery from anesthesia, and then returned to their home cages in order to avoid social stress.

### Intracerebroventricular injection

Rats were anesthetized with chloral hydrate (400 mg/kg, i.p.) and placed in a stereotaxic device. After the skull was exposed, a hole was drilled on the skull and a 26-gauge needle was inserted into the left lateral ventricle at coordinates: −0.4 mm from the Bregma, 1.4 mm mediolateral, and 3.8 mm dorsoventral. Then, 5.0 μl of Notch pathway inhibitor DAPT (Sigma), dissolved in PBS containing 5% DMSO at a concentration of 8.3 mg/mL, was injected over a period of 10 min (0.5 μl/min). After injection, the needle was withdrawn slowly over 5 min. The control without DAPT was carried out in a similar manner. To study the effect of NDRG2 on the progenitor cells in the normal rat brain, 5.0 μl of NDRG2-overexpressing adenoviruses or adenoviral blank vectors was injected intracerebroventricularly described above. Seven days later, rats were sacrificed and their tissues were subjected to immunohistochemistry.

### Immunohistochemistry

Brain tissues were prepared as described previously (Shi et al., 2013; Zhang et al., 2016). Thirty-μm-thick coronal sections through the stab wound were cut by a cryostat (Leica CM 1950, Germany). After blocked with 5% normal goat serum in PBS plus 0.5% Triton X-100 for 1 h, sections were incubated with following appropriate primary antibodies diluted in blocking solution overnight at 4°C: rabbit polyclonal antibody against glia fibrillary acid protein (GFAP, 1: 1,000, Abcam, Cambridge, UK), rabbit monoclonal antibody against IbaI (1: 300, Abcam), rabbit polyclonal antibody against Ki67 (1: 500, Abcam), rabbit polyclonal antibody against NDRG2 (1: 300, Abnova, Taipei, Taiwan, China), mouse monoclonal antibody against Nestin (1: 500, Abcam), and rabbit polyclonal antibody against NeuN (1: 500, Abcam). The day after, sections were washed and incubated with the FITC- or TRITC-conjugated anti-rabbit/mouse IgG (1:400; Jackson ImmunoResearch, West Grove, PA, USA) in the blocking solution for 2 h at room temperature. After washed, sections were stained with Hoechst33342 (Sigma Aldrich, St Louis, MO, USA) for 5 min. Immunostaining signals were observed, and images were taken under a confocal laser scanning microscope (Fluoview FV300, Olympus, Tokyo, Japan). For cell counting, the numbers of NDRG2 and Nestin double-positive cells were counted in eight 0.16 mm^2^ rectangles (0.4 mm × 0.4 mm) around the stab wound by using Image-J (v1.43, NIH software, Bethesda, MD, USA). The immunoreactivity of Nestin was also quantified using Image-J (NIH software). NDRG2- and Nestin-positive cells in brain sections were counted in every three sections.

### Western blotting

Brain tissues containing the stab wounded region, or the SVZ were collected, and total proteins were extracted in a lysis buffer, containing 1% NP40, 0.5% sodium deoxycholate, 0.1% sodium dodecyl sulfate, 0.25 mM phenylmethylsulphonyl fluoride, 5 mg/ml aprotinin, and 1 mM sodium orthovanadate. After electrophoresed on 10% SDS-polyacrylamide gels, proteins were transferred onto nitrocellulose membranes, which were incubated appropriate primary antibodies overnight at 4°C: anti-β-actin (Santa Cruz Biotechnology, Santa Cruz, CA, USA), anti-GFAP (1: 2,000, Abcam), and Hes1 (1: 1,000, Abcam), anti-NDRG2 (1: 1,000, Abnova), anti-Nestin (1: 1,000, Abcam), or anti-activated Notch1 (NICD, 1: 1,000, Abcam). For detection, horseradish peroxidase-conjugated secondary antibodies (Cell Signaling Technology) and an enhanced chemiluminescence system (Amersham Biosciences, Piscataway NJ, USA) were used. For quantitation, the intensity of each band was quantified using Image-J software (NIH)., and then the data acquired were normalized to β-actin expression and further normalized to their corresponding controls.

### Statistical analysis

All the data were presented as mean ± SEM of at least three independent experiments. Most statistical analyses were taken by a one-way analysis of variance (ANOVA), followed by a Dunnett’s multiple comparison test. *P* < 0.05 was considered to be statistically significant.

## Results

### Exclusive expression of NDRG2 in astrocytes in cortical parenchyma

**FIGURE 1:**
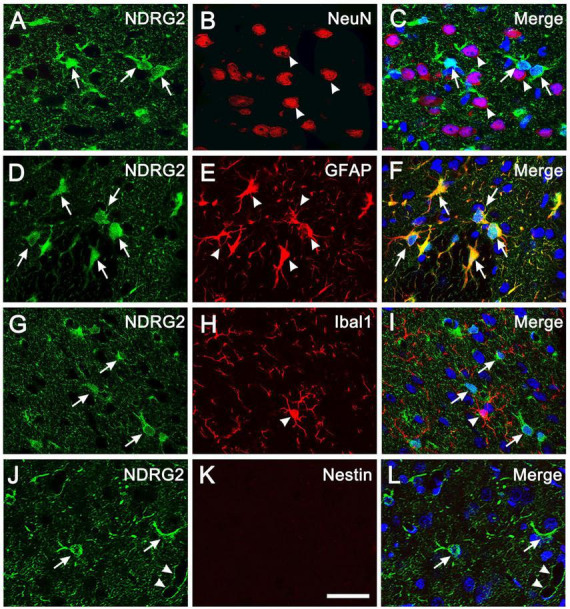
NDRG2 is exclusively expressed in astrocytes in rat cortical parenchyma. Immunostaining for NDRG2 (green) and cellular markers (red) including NeuN, GFAP, Iba1, and Nestin, was performed on normal cortical sections in rats. **(A–I)** NDRG2 was exclusively co-labeled with GFAP **(D–F)** but not with NeuN **(A–C)**, or IbaI **(G–I)**. **(J–L)** Nestin was not expressed in normal cortical parenchyma **(K)**. Cell nuclei were counterstained with Hoechst33342 (blue). Arrows show representative NDRG2-positive cells and arrowheads show representative NeuN-, GFAP- and Iba1-positive cells. Arrowheads in panel **(L)** show the immunoreactivity of NDRG2 surrounding blood vessels. Scale bar in panel **(K)**: 30 μm.

Immunohistochemical analysis showed that in the normal cortical parenchyma, NDRG2-positive cells were co-labeled with GFAP, a marker of astrocytes, but not co-labeled with NeuN and Iba1, markers for neurons and microglia, respectively, (Figures 1A-I; Supplementary Figure 2). These results were consistent with previous reports (Okuda et al., 2008; Flugge et al., 2014; Takarada-Iemata et al., 2014, 2018). In addition, NDRG2-positive cells did not express Nestin, a widely used marker of NSCs, which was absent in normal cortical parenchyma (Figure 1K; Supplementary Figure 2). NDRG2 expression was observed in the cytoplasm of astrocytes but not in the nuclei (Figures 1D-F). NDRG2 immunoreactivity was also observed in the surrounding blood vessels (Figures 1J, L, arrowheads; Supplementary Figure 2), probably indicating astrocytic end-feet adjacent to capillaries. These results showed that NDRG2 was exclusively expressed in astrocytes in the cortical parenchyma of normal rats.

### Increased NDRG2 expression in Rad-PCs after cortical stab injury

**FIGURE 2:**
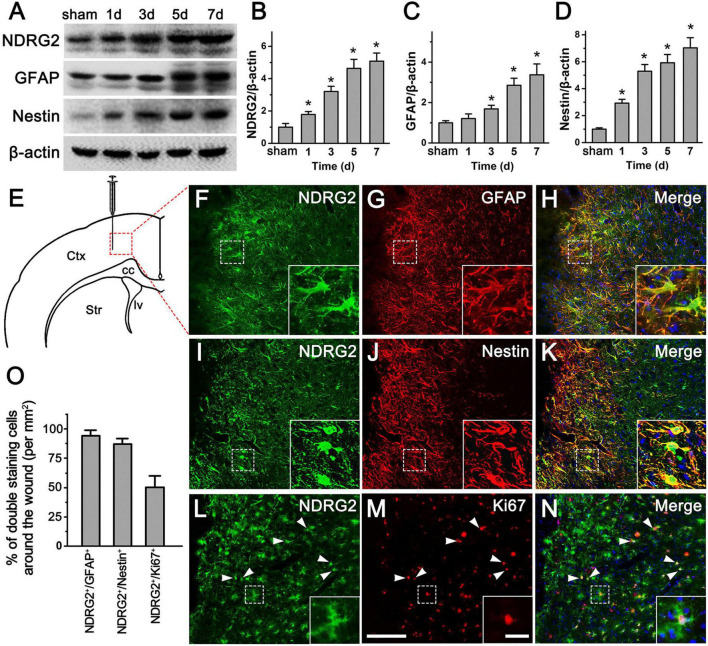
NDRG2 expression is increased in Rad-PCs after brain stab injury. **(A–D)** Immunoblotting analysis showed that at 1, 3, 5, and 7 day following stab injury, the levels of NDRG2, GFAP, and Nestin proteins around the wound were gradually increased with time. β-actin was used as an internal control. Quantified data are presented in panels **(B–D)**. Data are shown as mean ± SEM (*n* = 6). *p < 0.05, vs. the sham control. **(E)** Schematic illustration of brain section from wounded area. Tissue was dissected from the area delineated by the dotted box. Ctx, cortex; cc, corpus callosum; lv, lateral ventricle; Str, striatum. **(F–N)** At 7 day after stab injury, most of NDRG2-expressive cells (green) were co-labeled with GFAP (red), **(F–H)** and Nestin (red), **(I–K)**, and some of NDRG2-positive cells expressed cell proliferative marker Ki67 (red), **(L–N)**. Cell nuclei were counterstained with Hoechst33342 (blue). Arrowheads show representative NDRG2/Ki67-positive cells. Insets represent the higher magnification of dotted boxes. Scale bar in panel **(M)**: 100 μm and in inset: 20 μm. **(O)** The percentages of NDRG2^+^/GFAP^+^, NDRG2^+^/Nestin^+^, and NDRG2^+^/Ki67^+^ cells were qualified.

In the adult brain, astrocytes could become reactive and acquire NSC properties following traumatic injury (Buffo et al., 2008; Shimada et al., 2012; Gotz et al., 2015). To investigate the potential involvement of NDRG2 in traumatic injury, we assessed the change in expression of NDRG2 in injury-induced activated glial cells in rat cortex in a stab injury model. Following the injury, increased NDRG2 expression around the wound area was observed as early as day 1, and continued till reaching a peak at 7 day, as compared with the sham control (Figures 2A, B and Supplementary Figure 3). Compared to that of NDRG2, the expression of GFAP, a marker for rest and reactive astrocytes, remained unchanged at 1 day, began to increase at 3 day and then increased further at 5 and 7 day (Figure 2C). Similar to that of NDRG2, the expression of Nestin, a marker for both NSCs and Rad-PCs, was also shown to gradually increase from 1 to 7 day post injury (Figure 2D). Immunohistochemical results showed that at 7 day following injury, the high expression levels of NDRG2 and GFAP were observed in the region around the wound area, and most of NDRG2-expressing cells (95.6%) were co-labeled with GFAP (Figures 2F-H, O and Supplementary Figure 4). Additionally, the expression of Nestin was markedly increased in the area around the wound even though it is usually not expressed in the normal cortex (Figure 1K) or in the region far away from injured area (data not shown). Importantly, most of Nestin-expressing cells (90.9%) were co-labeled with NDRG2 (Figures 2I-K, O and Supplementary Figure 4). Moreover, a number of NDRG2-positive cells (54.8%) adjacent to the wounded site expressed the cell proliferation marker Ki67 (Figures 2L-N, O and Supplementary Figure 4). Taken together, these data revealed that NDRG2 was upregulated in response to traumatic injury and was expressed in the Rad-PCs.

### Regulatory effects of NDRG2 on the number of Rad-PCs

**FIGURE 3:**
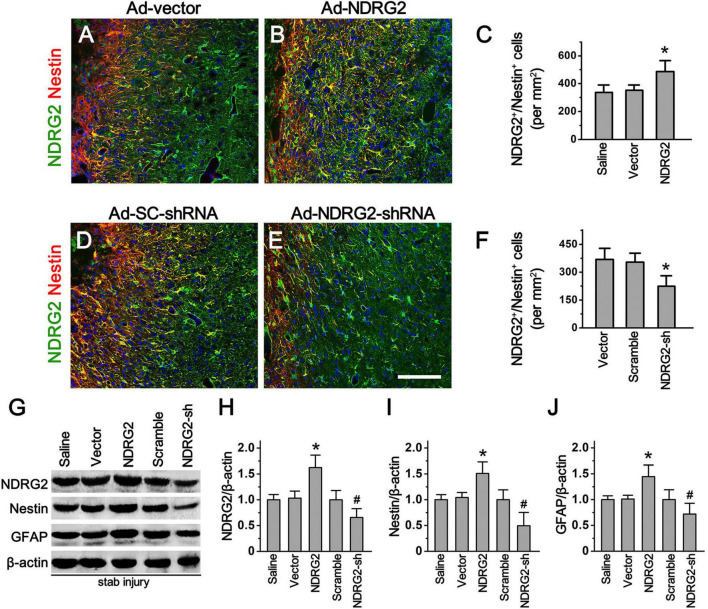
NDRG2 controls the formation of Rad-PCs. **(A–F)** At 7 day after stab injury, double immunostaining of NDRG2 (green) and Nestin (red) was performed. Adenovirus-mediated NDRG2 overexpression (Ad-NDRG2) significantly elevated the number of NDRG2^+^/Nestin^+^ cells around the wound **(B)**. Quantified data are presented in panel **(C)**. Data are shown as mean ± SEM (*n* = 5). *p < 0.05, vs. the blank vector (Ad-vector) and saline control. **(D–F)** Knockdown of NDRG2 expression by adenovirus-based shRNA (Ad-NDRG2-shRNA) decreased the number of NDRG2^+^/Nestin^+^ cells. Quantified data are presented in panel **(F)**. Data are shown as mean ± SEM (*n* = 5). *p < 0.05, vs. the scramble (Ad-SC-shRNA) and blank vector control. Cell nuclei were counterstained with Hoechst33342 (blue). Scale bar in panel **(E)**: 100 μm. **(G–J)** Immunoblotting analysis showed that NDRG2 overexpression (NDRG2) upregulated the expression of NDRG2, Nestin and GFAP while knockdown of NDRG2 expression (NDRG2-sh) downregulated the expression of these factors. β-actin was used as an internal control. Quantified data are presented in panels **(H–J)**. Data are normalized to the saline control and shown as mean ± SEM (*n* = 4). *p < 0.05, vs. the vector control; ^#^*p* < 0.05, vs. the scramble control.

To further explore whether increased expression of NDRG2 induced by stab injury is responsible for the formation of Rad-PCs, we generated recombinant adenoviral *ndrg2* overexpression and knockdown (shRNA) constructs and injected the viral-based constructs into the wounded site immediately after stab injury. At 7 d after stab injury, immunohistochemical analysis showed that injection of the viral based NDRG2 overexpression construct increased NDRG2/Nestin double-labeling cells (486 ± 79/mm^2^) around the lesion site, compared with the saline control (338 ± 45/mm^2^) and blank vector control (352 ± 38/mm^2^) (Figures 3A–C; Supplementary Figure 5). In contrast, injection of the viral based NDRG2 shRNA knockdown construct decreased NDRG2^+^/Nestin^+^ cells (224 ± 57/mm^2^) as compared to the blank vector control (368 ± 57/mm^2^) and scramble control (354 ± 48/mm^2^) (Figures 3D–F; Supplementary Figure 5). Consistent with these results, overexpression of NDRG2 led to increased Nestin expression while knockdown of NDRG2 led to decreased Nestin expression. In addition, NDRG2 appeared to also positively regulate the expression levels of Nestin and GFAP (Figures 3G–J; Supplementary Figure 6). Thus, these results indicated that NDRG2 may play a role in the regulation of Rad-PCs proliferation in cortical parenchyma after traumatic injury.

### Involvement of Notch signal into NDRG2-regulated Rad-PCs

**FIGURE 4:**
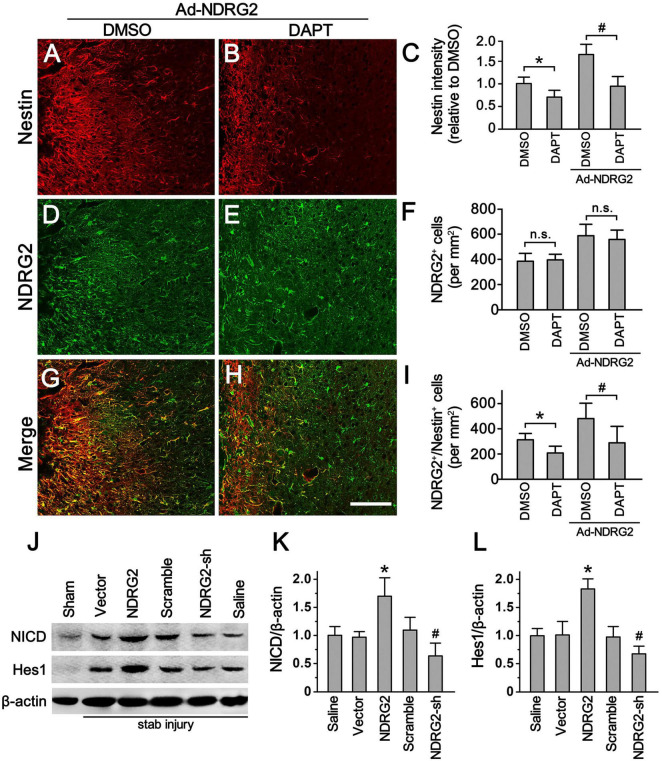
Notch signal involves in NDRG2-regulated Rad-PCs. At the time when the stab-injured rats were received with or without recombinant adenoviruses encoding *ndrg2* (Ad-NDRG2), they were treated with DAPT or DMSO intracerebroventricularly. **(A–I)** DAPT significantly decreased the immunoreactivity of Nestin **(A–C)** and the number of NDRG2^+^/Nestin^+^ cells **(G–I)** but did not affect the number of NDRG2^+^ cells **(D–F)**. Scale bar in panel **(E)**: 100 μm. Data are shown as mean ± SEM (*n* = 5). *p < 0.05, vs. the DMSO control. ^#^*p* < 0.05, vs. the DMSO control treated with NDRG2-expressing adenoviruses. n.s., no significance. **(J–L)** Immunoblotting analysis showed that NDRG2 overexpression (NDRG2) upregulated the expression of NICD and Hes1 while knockdown of NDRG2 expression (NDRG2-sh) downregulated the expression of NICD and Hes1. In the controls not subjected to stab injury, only slight expression of NICD and Hes1 was observed. β-actin was used as an internal control. Data are normalized to the saline control and shown as mean ± SEM (*n* = 5). *p < 0.05, vs. the vector control; ^#^*p* < 0.05, vs. the scramble control.

Since Notch signaling pathway is known to play a key role in the regulation of neurogenesis in both developmental and adult mammalian brain (Engler et al., 2018; Zhang et al., 2018), we next investigated whether Notch signal was involved in NDRG2-regulated Rad-PCs as well. To accomplish this, we examined how NDRG2 affected Nestin expression and Rad-PCs in combination with the blockade of Notch signaling pathway. When stab-injured rats were received with or without recombinant adenoviruses encoding *ndrg2*, they were administrated intracerebroventricularly with DAPT, a gamma-secretase inhibitor which can block Notch signaling pathway, or with DMSO as the control. At 7 day following injury, compared with the control, DAPT significantly decreased the immunoreactive signals of Nestin (*P* < 0.05, Figures 4A–C; Supplementary Figure 7) and the number of Nestin^+^/NDRG2^+^ cells (*P* < 0.05, Figures 4G–I; Supplementary Figure 7), and however, did not alter the number of NDRG2-positive cells (*P* > 0.05, Figures 4D–F; Supplementary Figure 7). Moreover, overexpression and knockdown of NDRG2 by recombinant viruses upregulated and downregulated the expression of Notch intracellular domain NICD and Notch target gene Hes1, respectively, (Figures 4J–L; Supplementary Figure 8). Thus, these results suggested the possibility that Notch signal may be involved in NDRG2-regulated Rad-PCs. Additionally, the results that Notch signal inhibition attenuated NDRG2-elevated number of Rad-PCs but did not affect the expression of NDRG, implying that NDRG2 may act as an upstream effector of Notch signal in the process of regulating Rad-PCs.

### Regulatory effects of NDRG2 on endogenous NSCs in the SVZ

**FIGURE 5:**
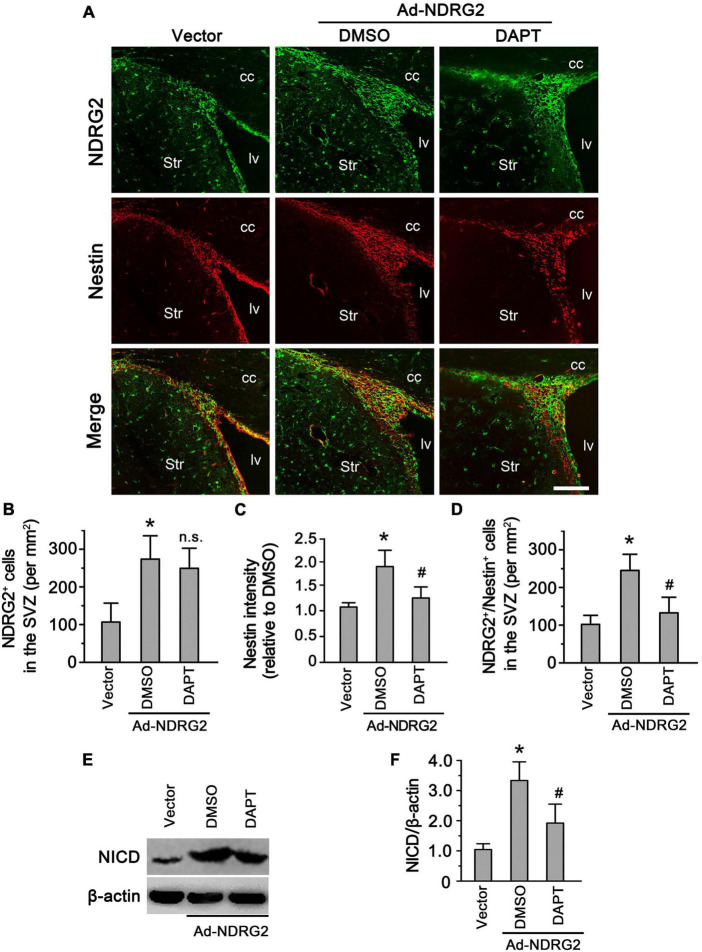
NDRG2 regulates endogenous NSCs in the SVZ *via* Notch signal. At the time when NDRG2-overexpressing adenoviruses were injected into the lateral ventricle, DAPT was administrated intracerebroventricularly into the contralateral ventricle. At 7 day following injection, double immunostaining of Nestin and NDRG2 was performed. **(A–D)** Compared with the blank vector control, NDRG2 overexpression significantly increased the immunoreactive signals of Nestin **(C)** and the number of Nestin^+^/NDRG2^+^ cells **(D)** in the SVZ. DAPT was able to mitigate these effects induced by NDRG2 overexpression compared with the DMSO control **(C,D)** but did not affect the number of NDRG2^+^ cells (n.s., no significance), **(B)**. cc, corpus callosum; lv, lateral ventricle; Str, striatum. Scale bar in panel **(A)**: 50 μm. **(E,F)** Under the normal condition, low levels of NICD were observed in the SVZ. Adenovirus-mediated NDRG2 overexpression significantly increased NICD expression of the SVZ and, however, DAPT attenuated NDRG2-induced NICD expression. Data are normalized to the DMSO control and shown as mean ± SEM (*n* = 5). *p < 0.05, vs. the blank vector control. ^#^*p* < 0.05, vs. the DMSO control.

As mentioned above, besides injury-induced Rad-PCs from brain parenchyma, another important type of NSCs are endogenous NSCs from neurogenic niches. To study whether NDRG2 also regulates endogenous NSCs under the normal condition, we injected NDRG2-overexpressing adenoviruses into rat lateral ventricle, and meanwhile DAPT was administrated intracerebroventricularly into the contralateral ventricle. At 7 day following injection, compared with the vector control, NDRG2 overexpression significantly increased the immunoreactive signals of Nestin (*P* < 0.05) and the number of Nestin^+^/NDRG2^+^ cells (*P* < 0.05) in the SVZ, a neurogenic niche generating endogenous NSCs (Figures 5A, C, D; Supplementary Figure 9). However, DAPT was able to mitigate these effects induced by NDRG2 overexpression (Figures 5A, C, D). It was noted that DAPT did not affect the number of NDRG2-positive cells (*P* > 0.05, Figures 5A, B). Furthermore, NDRG2 overexpression increased NICD expression of the SVZ, which can be attenuated by DAPT (*P* < 0.05, Figures 5E, F; Supplementary Figure 10). These results suggested that NDRG2 may regulate the number of endogenous NSCs in the SVZ under the normal condition *via* Notch signal as well.

## Discussion

In the present study, we demonstrated that NDRG2 was markedly upregulated in Rad-PCs after cortical stab injury, and may be involved in regulating their formation positively, and Notch signal may play an important role in this process. To our knowledge, this is the first evidence clarifying the role of NDRG2 in governing the formation of injury-induced progenitor cells in cortical parenchyma.

In the central nervous system, a group of studies have demonstrated that NDRG2 expression is predominantly present in GFAP-positive astrocytes in brain parenchyma under the physiological or pathological condition (Okuda et al., 2008; Takeichi et al., 2011; Flugge et al., 2014; Takarada-Iemata et al., 2014, 2018) and in dystrophic neurons in Alzheimer’s disease (Mitchelmore et al., 2004). Recently, we first provided definite evidence that *ndrg2* mRNA was also highly expressed in neural progenitor cells in the SVZ of the lateral ventricle and SGZ of the hippocampus in both embryonic and post-natal mouse brain (Liu et al., 2012), suggesting that NDRG2 was expressed in endogenous NSCs and may be involved in the regulation of neurogenesis. As an extending study, here we explore whether NDRG2 plays a role in injury-induced progenitor cells in the parenchyma of rat brain as well. We found that in the normal rat cortex, NDRG2 was exclusively expressed in astrocytes (Figure 1), consistent with previous studies above. However, when stab injury occurred, the cells around the wound expressed high level of NDRG2 as early as 1 day following injury, which was gradually increased with time. Importantly, most of these NDRG2-expressing cells expressed Rad-PC marker Nestin (Figure 2). Additionally, it was noted that the expression of NDRG2 and Nestin was significantly elevated as early as day 1 after injury, a time when GFAP upregulation did not occur yet (Figure 2). Therefore, the expression of NDRG2 or Nestin preceding that of GFAP implied that NDRG2 may regulate Rad-PCs just after stab injury even before astrocytes were activated.

To clarify whether this increased expression of NDRG2 was responsible for the neurogenesis of Rad-PCs, we took advantage of recombinant adenoviral vectors to alter the expression of NDRG2 in the cells around the wound. Our findings showed that overexpression of NDRG2 was able to increase the number of Nestin-positive cells and the level of Nestin protein. In contrast, knockdown of NDRG2 expression decreased Nestin expression. Furthermore, NDRG2-induced Nestin expression could be attenuated by DAPT, a Notch signal blocker, which, however, did not affect the number of NDRG2-positive cells (Figure 3). These data suggested that NDRG2 may play an important role in controlling the number of Rad-PCs by regulating Notch signaling pathway. To our knowledge, at present the relationship between NDRG2 and Notch signal has little been reported. It was noted that a study on the effect of NDRG2 on vertebral somitogenesis showed that the level of Notch1 mRNA was not changed in *ndrg2*^–/–^ mouse embryos at E10.5 (Zhu et al., 2012), suggesting that NDRG2 did not regulate the expression of Notch 1 directly.

It has been attracting a great deal of attention to study the mechanisms that control de-differentiation of reactive astrocytes into NSCs, since this *in situ* cell population may reprogram *in vivo* to enhance neurogenesis in cortical areas around the wound after various insults, such as trauma and stroke (Buffo et al., 2008; Shimada et al., 2012; Gotz et al., 2015). A series of evidence has shown that Notch signal plays a critical role in the proliferation and self-renewal of embryonic and adult NSCs (Androutsellis-Theotokis et al., 2006; Breunig et al., 2007; Borghese et al., 2010; Veeraraghavalu et al., 2010; Engler et al., 2018). A recent study also revealed that Presenilin 1-based Notch 1 signaling may control the generation, proliferation, and self-renewal of Rad-PCs isolated from the cortical peri-infarct areas after stroke (Shimada et al., 2012). Consistently, present study revealed that Notch signal may also be involved in the formation of Rad-PCs following cortical stab injury. Moreover, we showed that Notch signal may be regulated by a tumor suppressor NDRG2 (Figure 4). Given our results that Notch inhibition by DAPT affected the number of Nestin^+^ cells but not that of NDRG2^+^ cells, combining with the evidence that NDRG2 did not affect Notch 1 expression (Zhu et al., 2012), we proposed that NDRG2 may be act as an upstream effector or through a non-cell-autonomous mechanism to regulate Notch signal. To be sure, further studies need to be done to clarify this possibility.

When diverse insults occur in the brain, astrocytes are activated and become so-called reactive astrocytes (Sofroniew and Vinters, 2010). These reactive astrocytes are response to the brain injury in a heterogeneous manner with only a subpopulation of cells resuming proliferation and acquiring stem cell properties (White et al., 2010). It was reported that GLAST-expressing reactive astrocytes were able to dedifferentiate and form multipotent spheres *in vitro* (Buffo et al., 2008) in a brain stab injury model. Most of RC2-positive reactive astrocytes were found to be proliferated in a mouse cerebral stroke model (LeComte et al., 2015). Additionally, this subgroup of reactive astrocytes was found to differentially express several stem cell-associated proteins such as GFAP, Nestin, and Sox2 (Pekny and Pekna, 2004; Buffo et al., 2008; Shimada et al., 2010). Our present study showed that following brain stab injury, a number of NDRG2-expressing reactive astrocytes were proliferated as revealed by Ki67 immunostaining, suggesting that the subpopulation of NDRG2-positive reactive astrocytes might contain the cell of origin for injury-induced progenitor cells.

In conclusion, here we demonstrated that NDRG2 may play an important role in controlling the formation of Rad-PCs by regulating Notch signaling pathway after brain stab injury. Therefore, proliferating NDRG2-positive reactive astrocytes may provide a promising target cell population for *in vivo* reprogramming to enhance neurogenesis in cortical areas around the wound after TBI.

## Data availability statement

The original contributions presented in this study are included in the article/Supplementary material, further inquiries can be directed to the corresponding author.

## Ethics statement

Animal experiments were approved by the Fourth Military Medical University Animal Experimentation Committee and conducted under the ethical guidelines in compliance with the National Institutes of Health Guidelines for the Care and Use of Laboratory Animals.

## Author contributions

QZ and MS contributed to the conception of this study. QZ, RS, and MH contributed to the generation of research data. DF and RW helped in the immunoblotting experiments. MS analyzed the research data and prepared the manuscript. All authors contributed to the article and approved the submitted version.
