# Supplementary material for: NDRG2 regulates the formation of reactive astrocyte-derived progenitor cells via Notch signaling pathway after brain traumatic injury in rats
Source: Front Mol Neurosci. 2023 Apr 4;16:1149683. doi: 10.3389/fnmol.2023.1149683 (PMC10112515; doi:10.3389/fnmol.2023.1149683)

Supplementary Material

NDRG2 regulates the formation of reactive astrocyte-derived progenitor cells via Notch signaling pathway after brain traumatic injury in rats

Qinjun Zhang, Rui Shi, Minghua Hao, Dongyun Feng, Rui Wu, Ming Shi*

*** Correspondence:** Ming Shi, Email: biomidas@163.com

Supplementary Figures

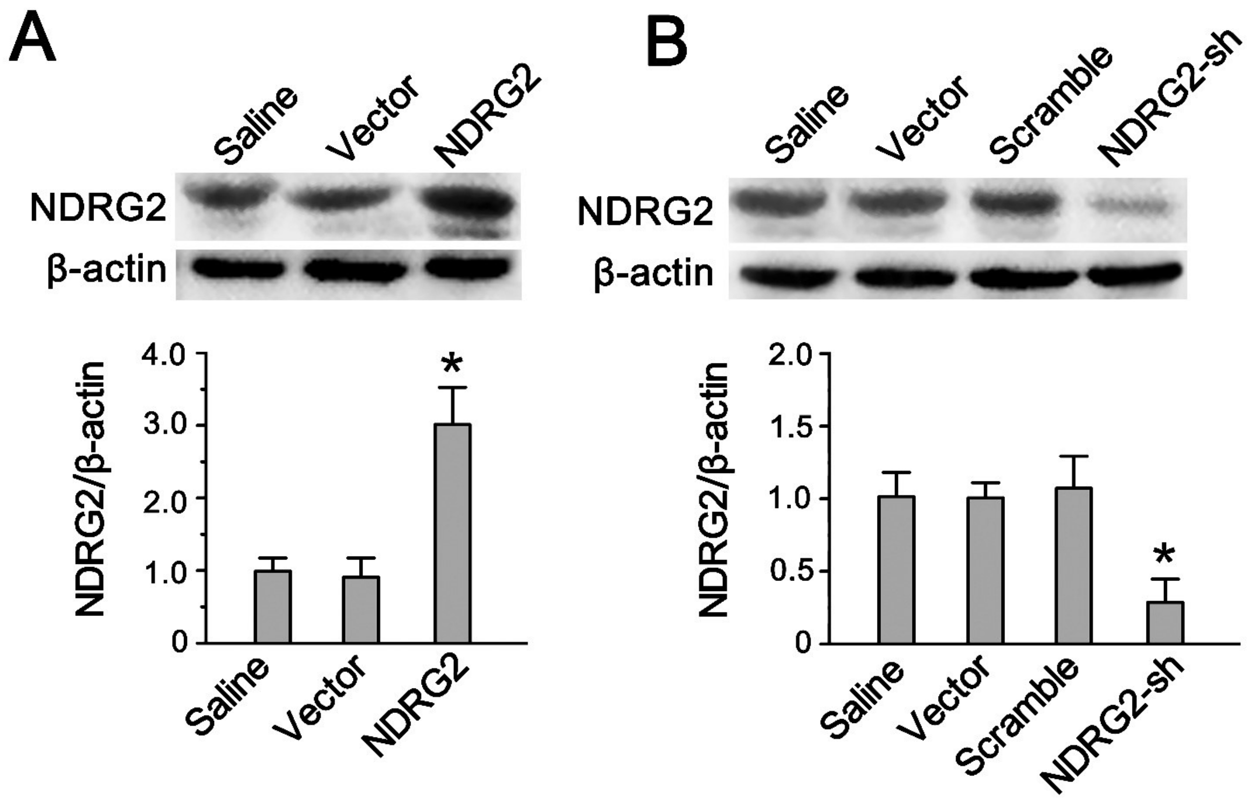

Supplementary Figure 1. The test of the efficiency of adenoviral-mediated upregulation or knockdown of NDRG2 expression. Adenoviruses encoding *ndrg2*, *ndrg2*-shRNA, *ndrg2*-scramble and blank vectors were amplified in HEK293 cells and then immunoblot analysis of NDRG2 was

performed. (A) Adenoviruses encoding *ndrg2* significantly upregulated the expression of NDRG2. (B) Adenoviral-mediated *ndrg2*-shRNA significantly downregulated the expression of NDRG2. Data are normalized to the saline control and shown as mean ± SEM (n = 4). *, $p < 0.05$, vs. the blank vector control (A) or the scramble control (B).

Supplementary Figure 2. Raw microscopy images for Figure 1(A-L)

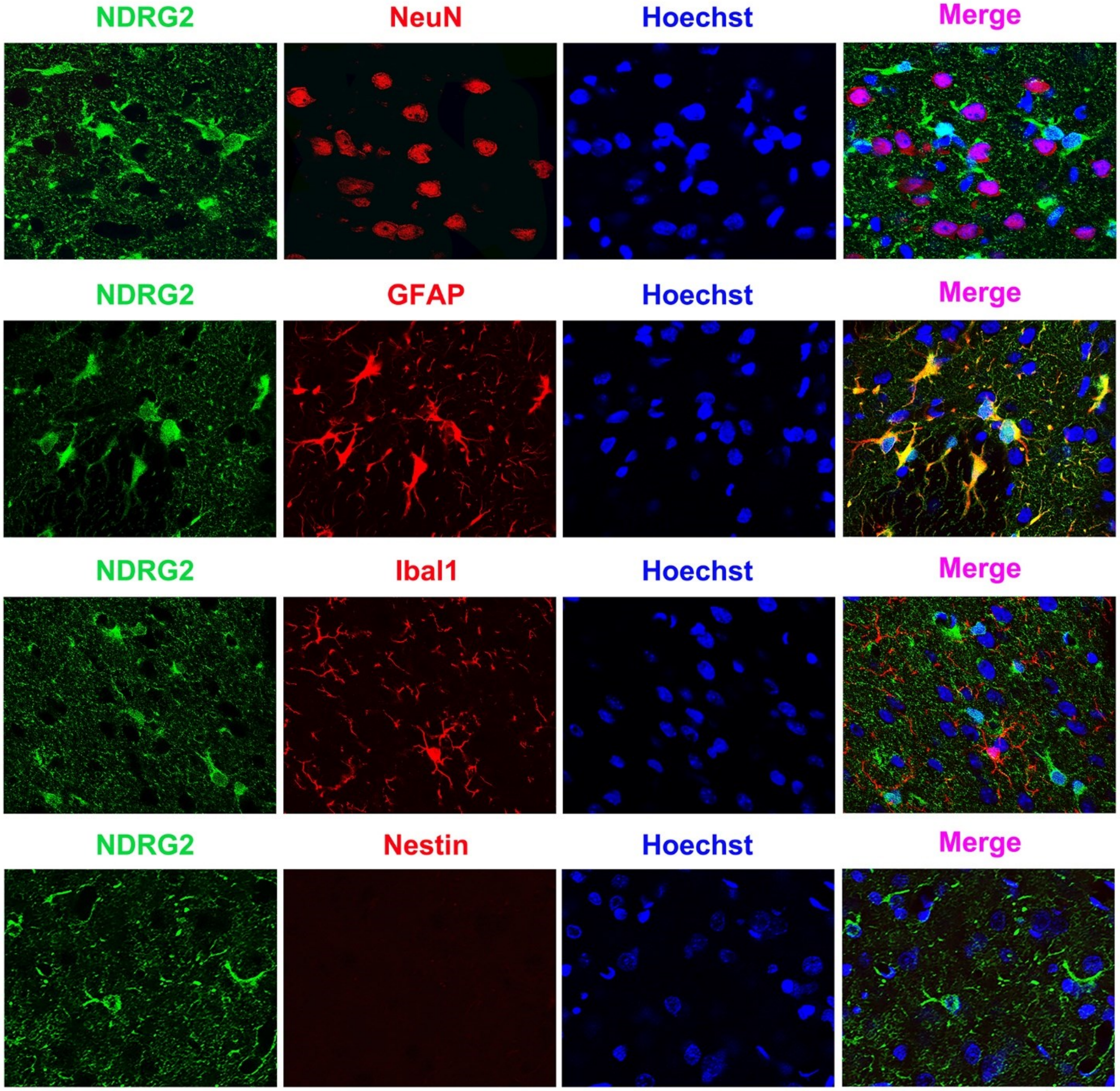

Supplementary Figure 3. Full-length blots for Figure 2(A)

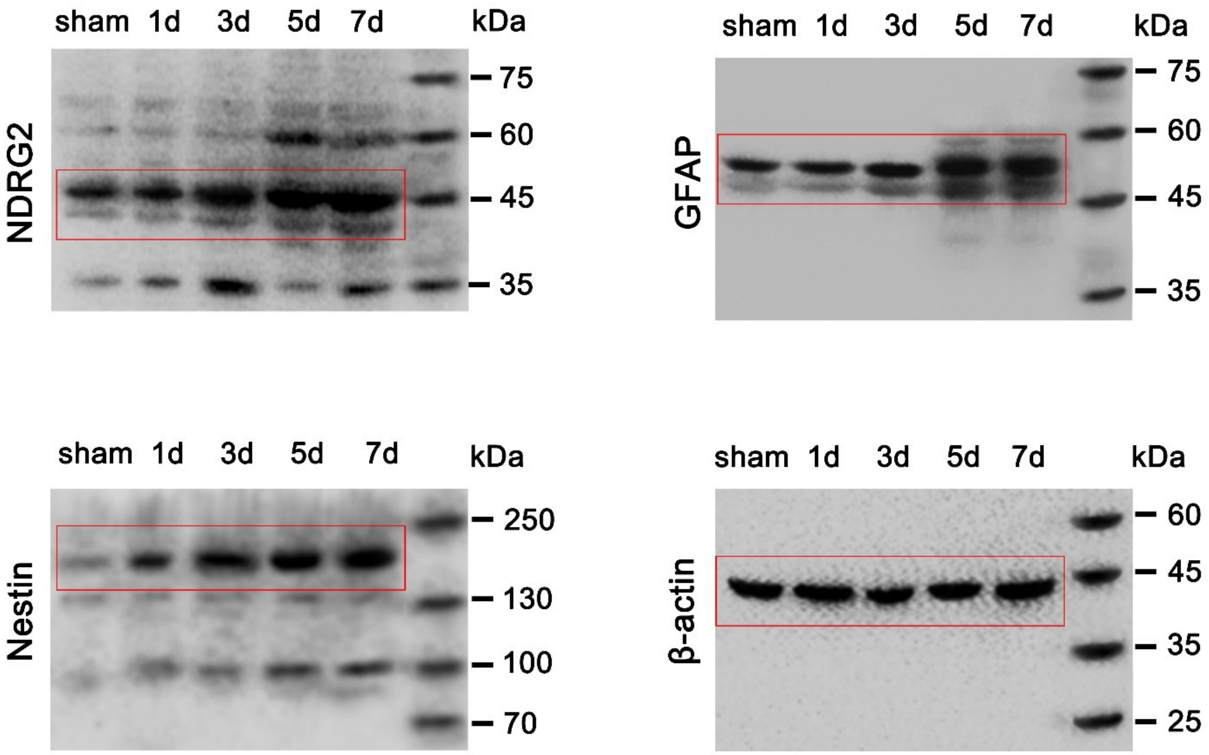

Supplementary Figure 4. Raw microscopy images for Figure 2(F-N)

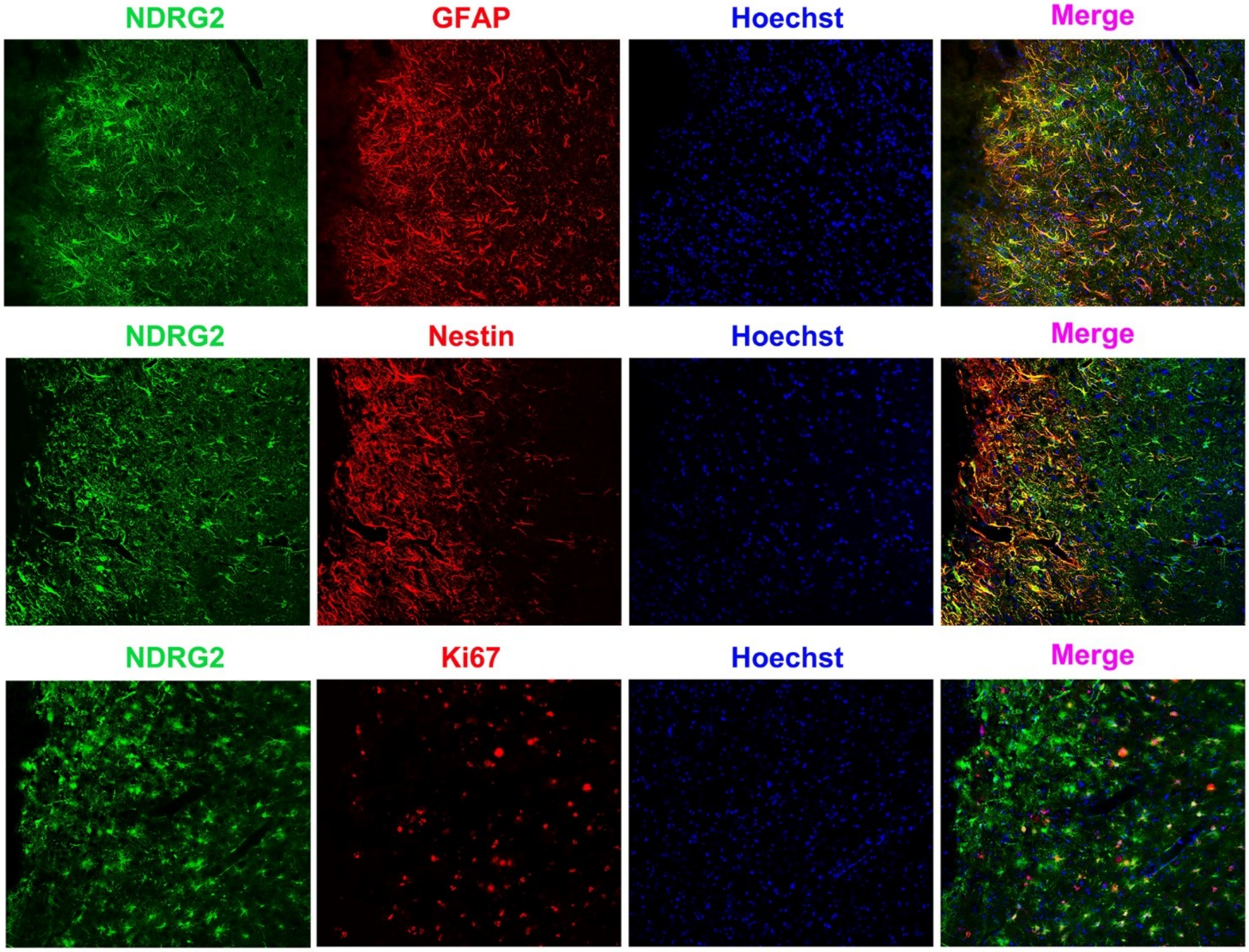

Supplementary Figure 5. Raw microscopy images for Figure 3(A, B, D, E)

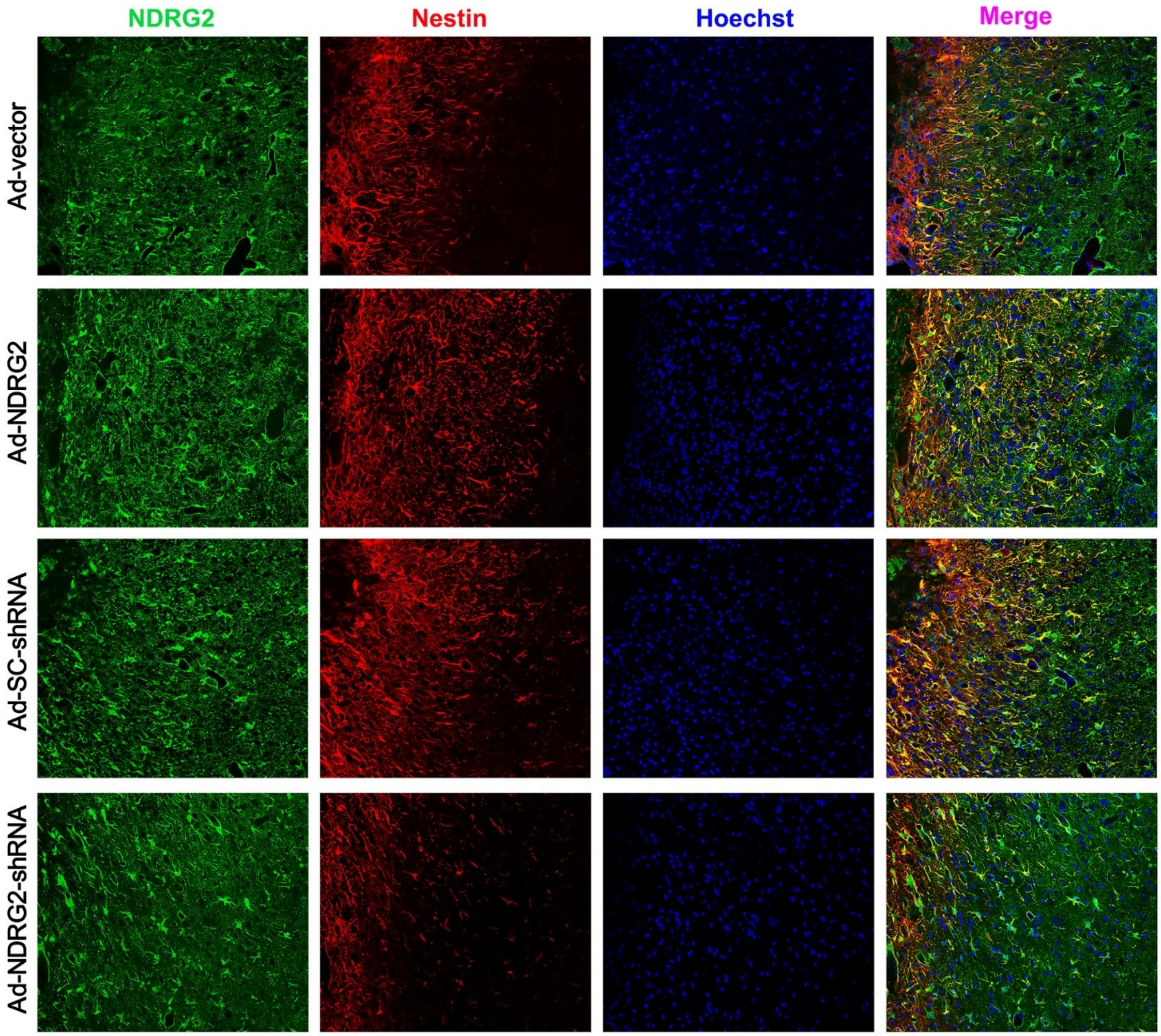

Supplementary Figure 6. Full-length blots for Figure 3(G)

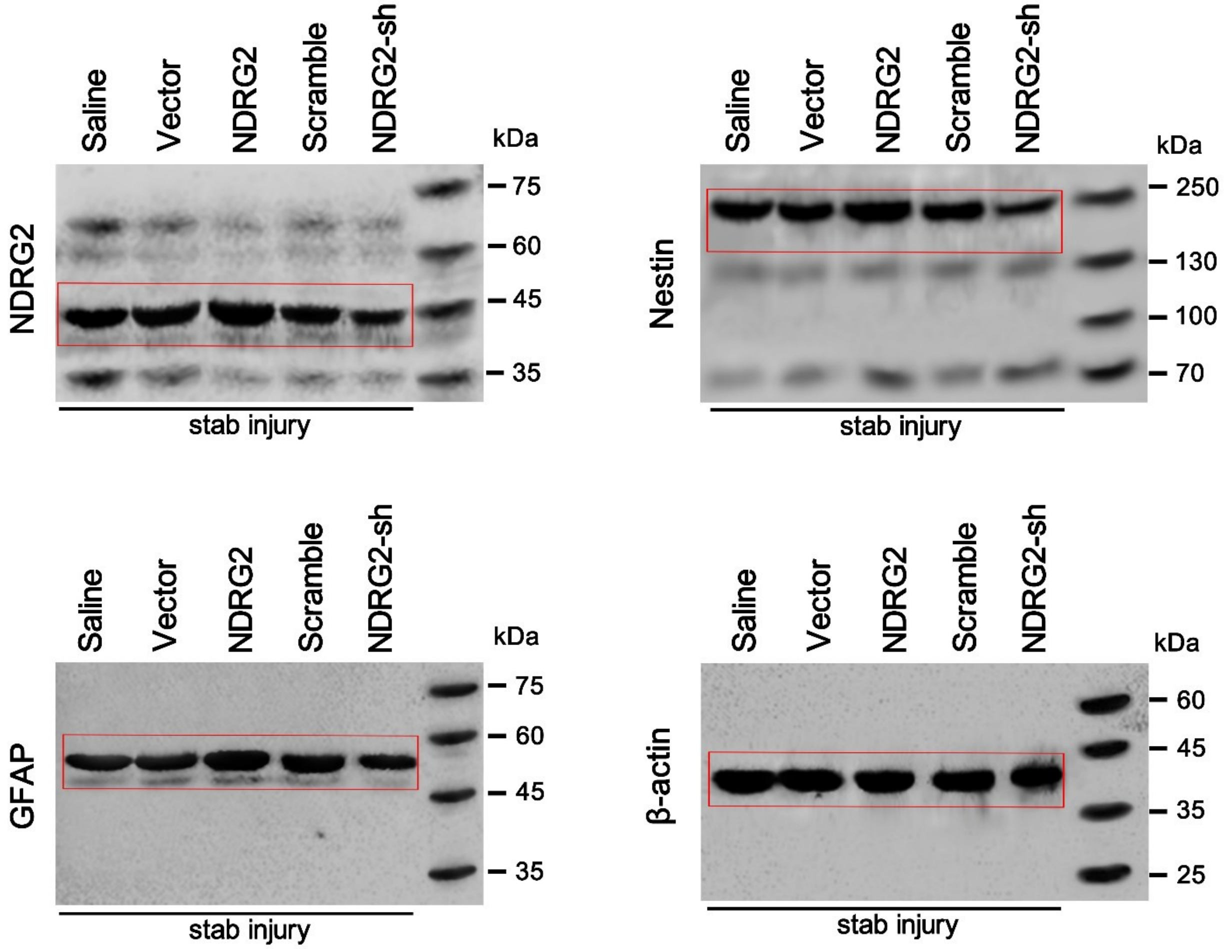

Supplementary Figure 7. Raw microscopy images for Figure 4(A, B, D, E, G, H)

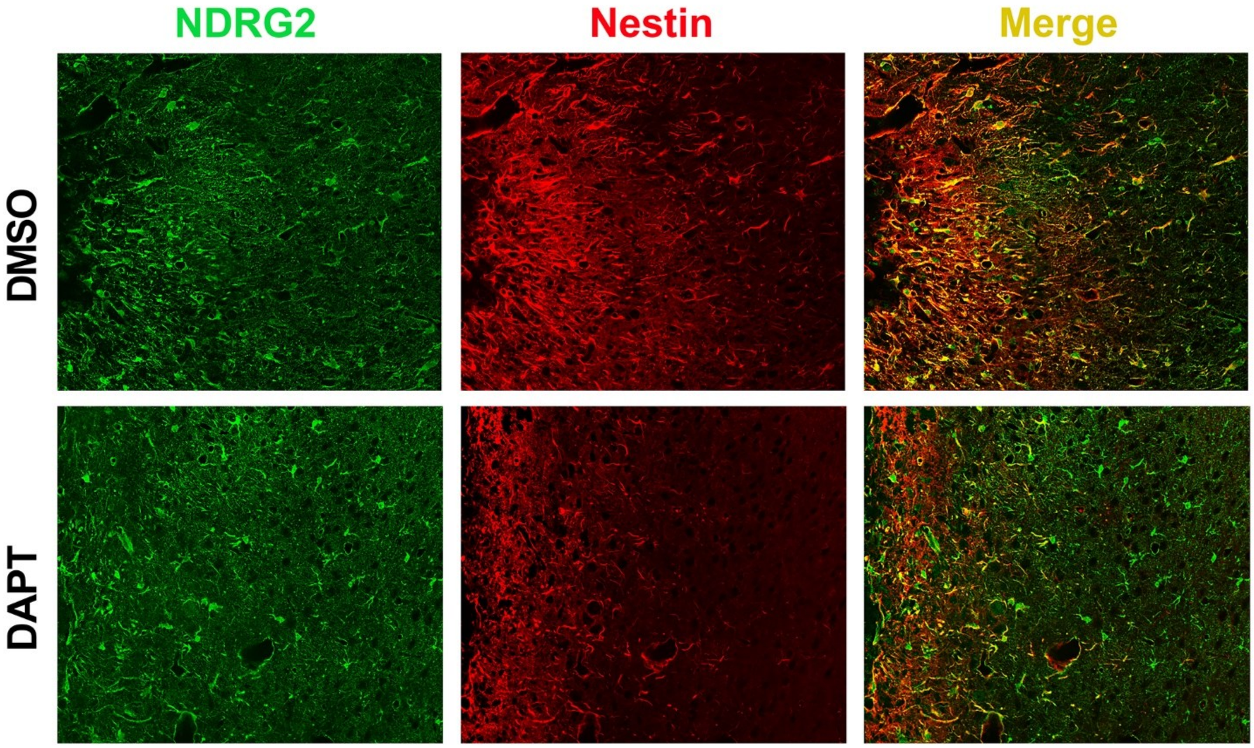

Supplementary Figure 8. Full-length blots for Figure 4(J)

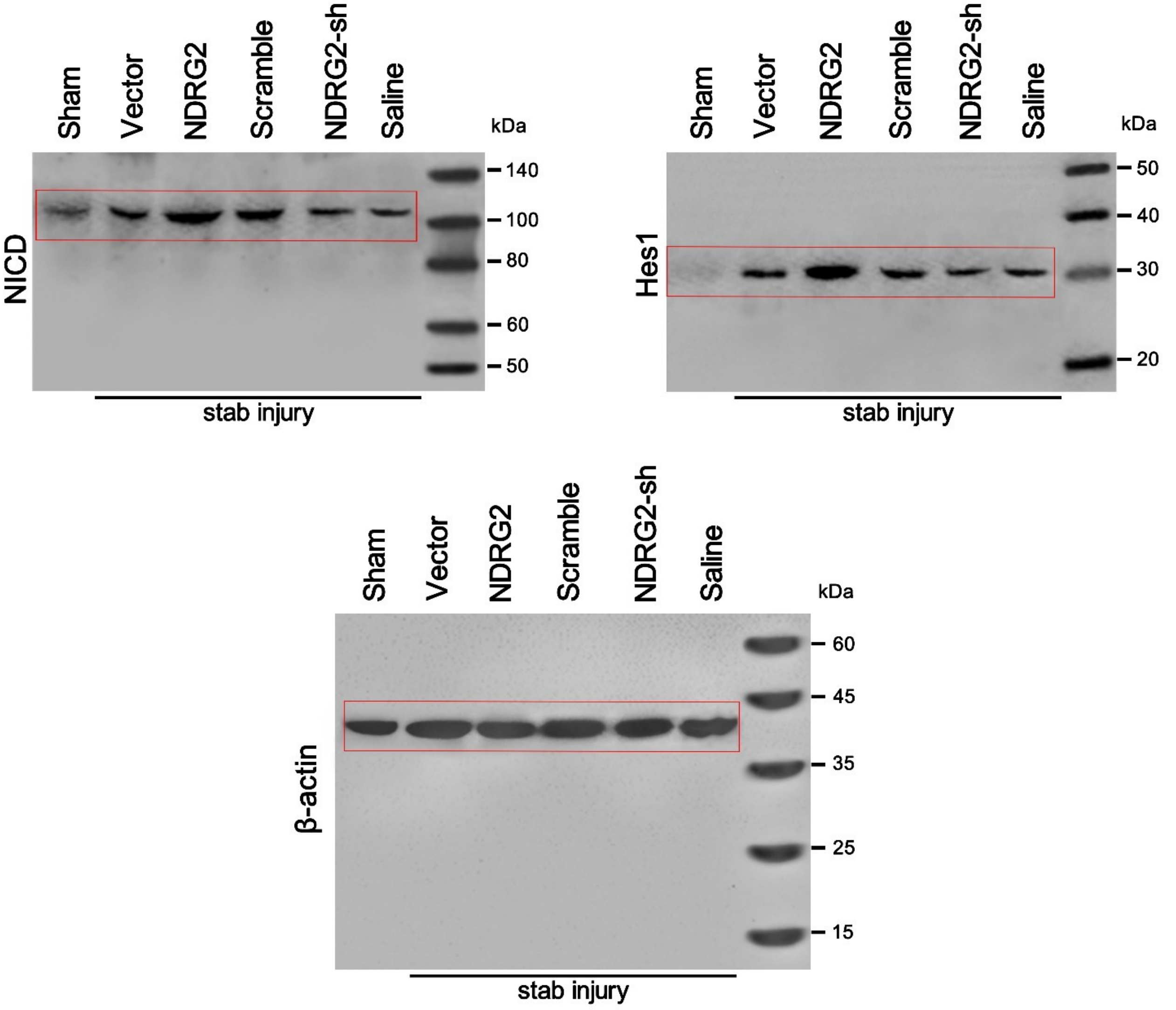

Supplementary Figure 9. Raw microscopy images for Figure 5(A)

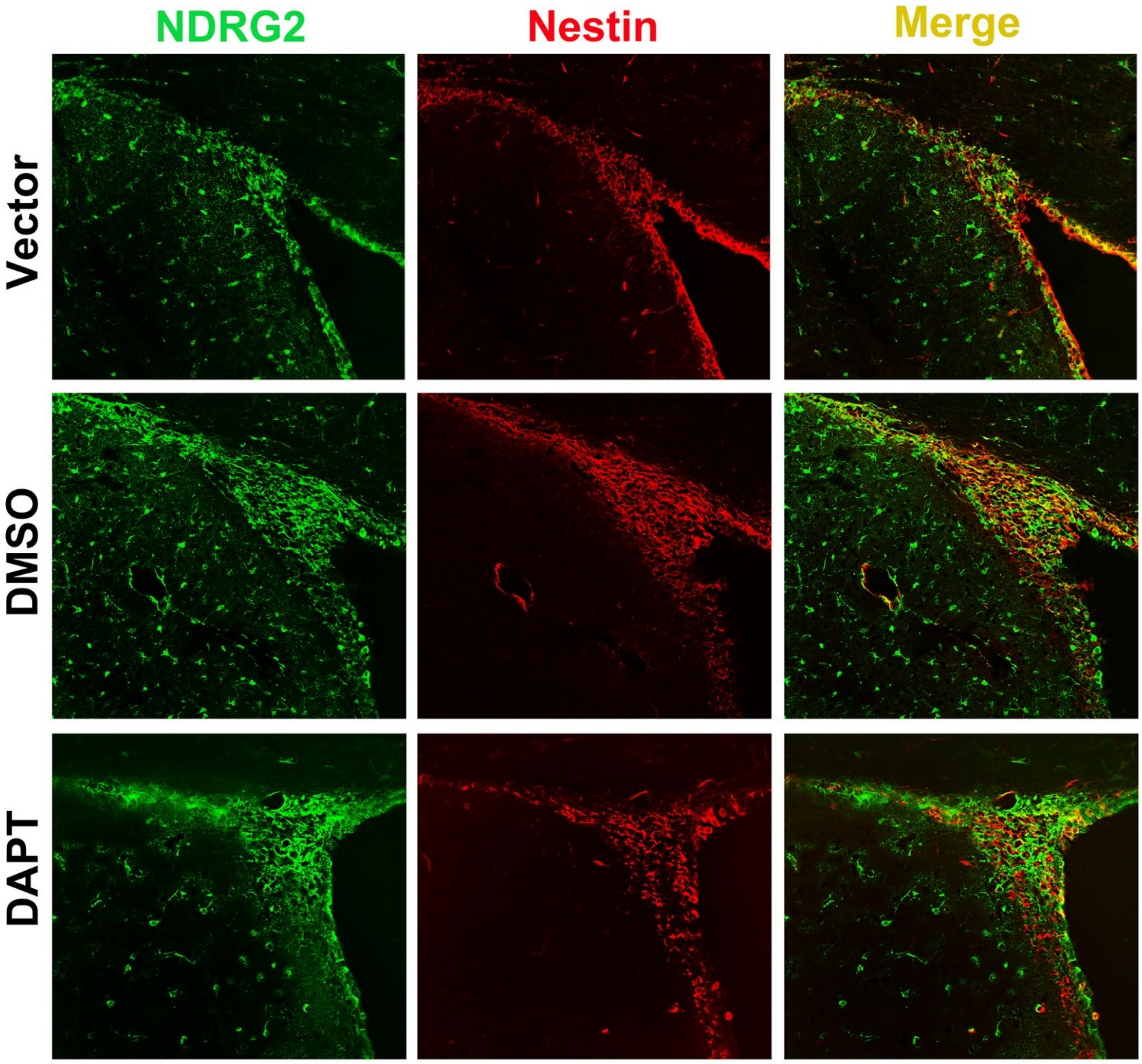

Supplementary Figure 10. Full-length blots for Figure 5(E)

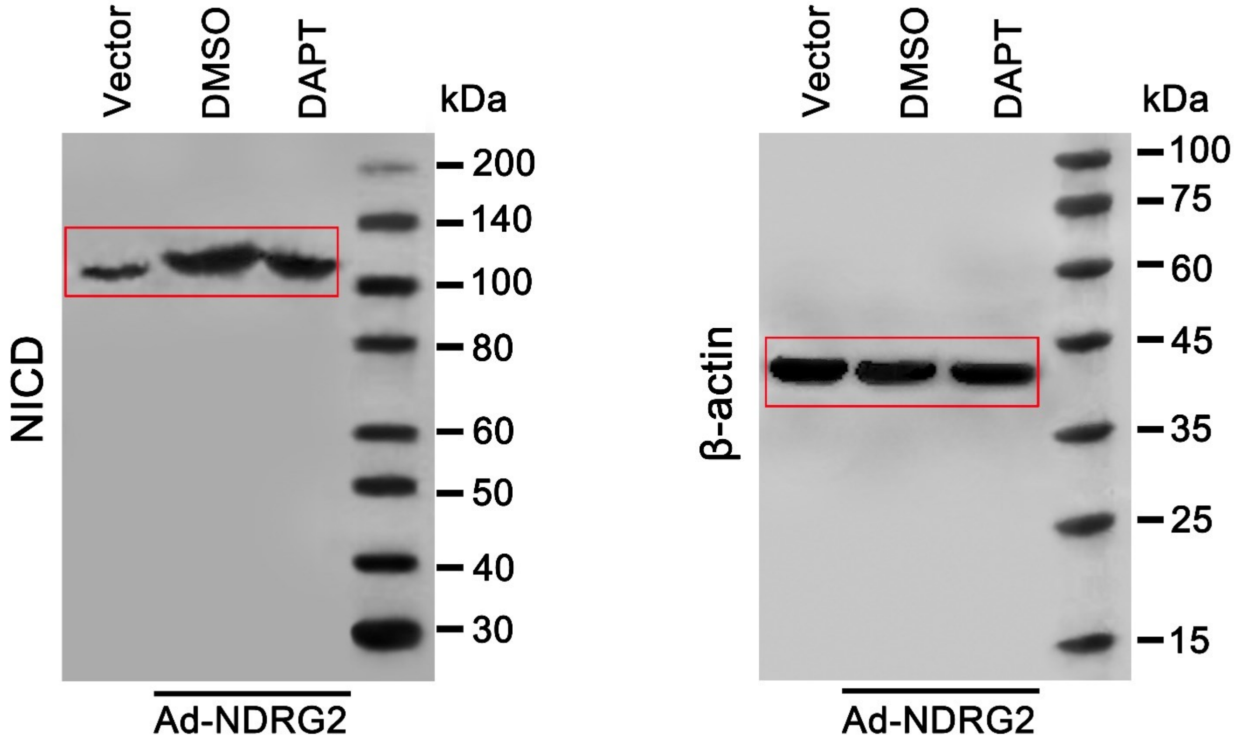

Supplement: Supplementary file 1 [file Data_Sheet_1.PDF]
